# Association of Genetic Markers in the BCL-2 Family of Apoptosis-Related Genes with Endometrial Cancer Risk in a Chinese Population

**DOI:** 10.1371/journal.pone.0060915

**Published:** 2013-04-23

**Authors:** Tsogzolmaa Dorjgochoo, Yong-Bing Xiang, Jirong Long, Jiajun Shi, Sandra Deming, Wang-Hong Xu, Hui Cai, Jiarong Cheng, Qiuyin Cai, Wei Zheng, Xiao-Ou Shu

**Affiliations:** 1 Division of Epidemiology, Department of Medicine and Vanderbilt-Ingram Cancer Center, Vanderbilt University School of Medicine, Vanderbilt University, Nashville, Tennessee, United States of America; 2 Department of Epidemiology, Shanghai Cancer Institute, Shanghai, China; College of Pharmacy, University of Florida, United States of America

## Abstract

**Background:**

*In vitro* studies have demonstrated the role of the BCL-2 family of genes in endometrial carcinogenesis. The role of genetic variants in BCL-2 genes and their interactions with non-genetic factors in the development of endometrial cancer has not been investigated in epidemiological studies.

**Patients and Methods:**

We examined the relationship between BCL-2 gene family variants and endometrial cancer risk among 1,028 patients and 1,922 age-matched community controls from Shanghai, China. We also investigated possible interactions between genetic variants and established risk factors (demographic, lifestyle and clinical). Individuals were genotyped for 86 tagging single nucleotide polymorphisms (SNPs) in the *BCL2, BAX, BAD* and *BAK1* genes.

**Results:**

Significant associations with endometrial cancer risk were found for 9 SNPs in the *BCL2* gene (*P* trend<0.05 for all). For SNPs rs17759659 and rs7243091 (minor allele for both: *G*), the associations were independent. The odds ratio was 1.27 (95% CI: 1.04–1.53) for women with *AG* genotype for the SNP rs17759659 and 1.82 (95% CI: 1.21–2.73) for women with the *GG* genotype for the SNP rs7243091. No interaction between these two SNPs and established non-genetic risk factors of endometrial cancer was noticed.

**Conclusion:**

Genetic polymorphisms in the *BCL2* gene may be associated with the risk of endometrial cancer in Chinese women.

## Introduction

Apoptosis is a selective process for deleting cells, which is an essential physiological process required for tissue size regulation and morphogenesis [1]. In mammalian cells, apoptosis is induced by two distinct signaling pathways: extrinsic or death receptor and intrinsic or mitochondrial [1], [2]. Proteins of the BCL-2 family have been identified as essential components of the mitochondrial pathway. Members of the BCL-2 family may promote or inhibit apoptosis by synthesizing anti-apoptotic (i.e., *BCL2, BCL-Xl*) or pro-apoptotic (i.e., *BAX*, *BAK, BAD, BID, BCL-Xs*) proteins [1]–[3]. Because of the cyclical pattern of *BCL2* expression in the normal endometrium during the menstrual cycle [4], [5], it has been suggested that the *BCL2* gene may be the most hormone-dependent member of the BCL-2 family of genes [4], [6].

BCL-2-family proteins play a crucial role in carcinogenesis. The pro-apoptotic *BAX, BAK* and *BAD* genes are believed to oppose cell carcinogenesis, while the *BCL2* gene can promote cancer cell growth by blocking apoptosis [7], [8]. Over expression of the anti-apoptotic *BCL2* gene has been observed in various human cancer tissues, including breast, colon, thyroid and endometrial carcinomas [7]–[10]. The relationship between the BCL-2 family of genes and endometrial cancer came to light mainly through *in vitro* studies of human tissue samples [6], [11], [12]. Over expression of *BCL2* slows down cell growth and very high expression can promote cell death, while lower *BCL2* expression can be a sign of inhibition of apoptosis in human breast and endometrial carcinoma tissues [13], [14]. Studies have also shown that *BCL2* expression differs by degree of tumor aggressiveness and differentiation [13]–[15], and *BCL2* expression has been shown to be very low or absent in higher grade carcinomas compared with lower grade carcinomas [14]. It has been postulated that *BCL2* expression may be suppressed during cancer progression [12], [16]. Thus, *BCL2* expression could be a valuable predictor of cancer progression and prognosis [17]–[19].

Only a few observational studies have investigated the relation of genetic variants in the BCL-2-gene family, particularly the *BCL2* gene, with susceptibility to myeloid leukemia, squamous cell carcinoma of the head and neck, esophageal cancer and prostate cancer [20]–[23]. The results suggest that the BCL-2 family of genes play an important role in cancer development. To date, no genetic association studies have been published on the role of BCL-2-gene family variants in the development of endometrial cancer. We investigated whether genetic variants in the BCL-2-family genes *BAD, BAX, BCL2* or *BAK1* are associated with endometrial cancer risk. Furthermore, we examined whether variants in these genes modify the effect of established non-genetic risk factors for endometrial cancer by using data from the Shanghai Endometrial Cancer Genetics Study (SECGS).

## Materials and Methods

### Ethics statement

The study protocols were approved by the Institutional Review Boards of participating institutes, i.e., the Vanderbilt University School of Medicine, Vanderbilt University, Nashville, Tennessee, United States; and the Shanghai Cancer Institute, Shanghai, China; Shanghai, China. All participants provided written, informed consent.

### Study population and data collection

The SECGS uses resources from two studies and includes subjects who participated in the Shanghai Endometrial Cancer Study (SECS) and control subjects who participated in the Shanghai Breast Cancer Study (SBCS, Phase 1). Both the SECS and SBCS are population-based, case-control studies conducted in Shanghai, China between 1996 and 2003, which used nearly identical study protocols. Details of the study designs for these two studies have been described in detail elsewhere [24], [25]. Briefly, 1,208 cases aged 30–69 years newly diagnosed with endometrial cancer were identified through the population-based Shanghai Cancer Registry between January 1997 and December 2003. Cancer diagnoses were confirmed by pathologists. Controls were randomly selected from the general population using the Shanghai Resident Registry and age-frequency matched to cancer cases. The current study includes 1,000 controls from the SECS and additional 922 cancer-free controls from the SBCS.

Study participants were interviewed to obtain detailed information on demographics, lifestyle habits, dietary intakes and supplement use, menstrual and reproductive history, hormone use, disease history, weight history and family history of any cancer. Anthropometric measurements, including weight, height and circumferences of the waist and hips, were taken by the interviewers. Menopause was defined as the cessation of the menstrual period for at least 12 months before the reference date (diagnosis date for cases and interview date for controls), excluding lapses caused by pregnancy, breastfeeding or estrogen hormone use. Body mass index (BMI, weight in kilograms divided by height in meters squared, kg/m^2^) and waist-to-hip circumference ratio (WHR) were calculated by using measured anthropometrics as described previously [26], [27].

### SNP selection, identification and genotyping

Haplotype-tagging SNPs (tagSNPs) in BCL-2-family genes were selected from the Han Chinese data of the International HapMap Project (http://hapmap.ncbi.nlm.nih.gov/) by using the Tagger program [28]. tagSNPs were selected based on the following criteria: 1) genotype call rate ≥95%, 2) minor allele frequency (MAF) ≥0.05, 3) located within a region starting 5 Kb upstream of the transcription start site and ending 5 Kb downstream of the stop codon of each gene and 4) linkage disequilibrium (LD) of *r^2^*≥0.9. SNPs with a known or potential function were all included. Genotyping was conducted at the Vanderbilt Microarray Shared Resource. As a quality control (QC) procedure, we included 39 blinded duplicate samples and 12 HapMap DNA samples in the genotyping. The average consistency rate for these samples was 99.6%. The laboratory staff members were blinded to the case-control status and identity of all samples. A total of 86 SNPs in BCl-2-family genes (72 SNPs in *BCL2*, 4 SNPs in *BAD*, 5 SNPs in *BAX*, and 5 SNPs in *BAK1*) were included in the study, with an average call rate of 99.8%.

### Statistical analyses

We used SAS software (version 9.2; SAS Institute, Inc.) for the statistical analyses. Demographic, lifestyle and clinical factors were compared between cases and controls by using the χ^2^ test for categorical variables and a *t*-test for continuous variables. Calculation of allele frequencies and testing for Hardy-Weinberg Equilibrium (HWE) were based on control data. LD between polymorphisms in the *BCL2, BAD, BAX*, and *BAK1* genes was assessed using HaploView, version 4.2 software [29]. Odds ratios (OR) and 95% confidence intervals (CIs) were derived from multivariate logistic regression models to evaluate the associations of the score of established risk factors with endometrial cancer risk and associations of cancer risk with genotypes [i.e., homozygous (*AA*) for the major allele, heterozygous (*Aa*) and homozygous (*aa*) for the minor allele] under additive, dominant and recessive genetic models. Age (continuous) and education (categorical) were adjusted for in all analyses. Additional adjustment for menopausal status, family history of endometrial cancer in first-degree relatives and BMI did not alter the gene-disease associations.

We derived risk scores for established risk factors for endometrial cancer based on previous literature [30]–[34] and their relative importance in our population (**Table 1**). Menopausal status was highly correlated with age, and smoking and hormone replacement therapy (HRT) use were not significantly associated with endometrial cancer risk in this population; thus, these factors were not included in the risk score calculation for established risk factors. As shown in Table 1, we assigned a numeric score (e.g., 0, 1, 2) to each category of the 9 risk factors that were associated with endometrial cancer in our population based on their contribution to total risk; these included age (years, <45 = 0; 45–54 = 1, ≥55 = 2); BMI (<18.5 = 0, 18.5–22.9 = 1, 23.0–27.4 = 2, ≥27.5 = 3); parity (nulliparous = 2, 1 = 1, ≥2 = 0), menstruation span (years: <28.0 = 0, 28.0–31.9 = 1; 32.0–35.1 = 2; ≥35.2 = 3), use of oral contraceptives (OC; never = 1, ever = 0), regular physical activity (never = 1, ever = 0), alcohol consumption (never = 1, ever = 0), history of diabetes or hypertension (no = 0, yes = 1) and history of endometrial or colorectal cancer in first-degree relatives (no = 0, yes = 1). For alcohol consumption and OC use we switched the scores (ever = 0 and never = 1), since these are protective factors for endometrial cancer risk based on the literature [30]–[34]. Menstruation span was calculated based on the difference between ages at menopause and menarche with consideration of pregnancy-related variables (breastfeeding and pregnancy history) and was categorized based on the quartile distribution among controls. BMI was categorized based on the World Health Organization (WHO) BMI cut-off points for Asian populations. For each participant, the sum of scores for all risk factors was calculated. Summary scores ranged from 0 to 14 and were used for the current analysis. We evaluated associations between endometrial cancer risk and scored established risk factors as continuous or categorical variables (based on tertile and median distributions among controls).

**Table 1:** Associations of endometrial cancer with established risk factors, the Shanghai Endometrial Cancer Genetics Study (SECGS), 1996 to 2003.

Established risk factors	Cases (*N* = 1,028) %	Controls (*N* = 1,922) %	Endometrial cancer risk OR (95% CI)*	*P* trend or *P* value	Risk score
Age, y (mean ± SD)	54.8±8.5	50.7±9.5		<0.01	-
<45	11.3	28.4	1.00 (reference)		0
45–54	41.4	37.6	2.81 (2.22–3.55)		1
≥55	47.3	34.1	3.67 (2.86–4.70)	<0.01	2
Education					
≤Elementary school	21.7	17.6	1.00 (reference)		
Middle school	38.2	40.8	1.50 (1.18–1.91)		
≥High school	40.1	41.6	1.48 (1.17–1.88)	0.008	
BMI, kg/m^2^					
<18.5	1.8	5.2	1.00 (reference)		0
18.5–22.9	23.4	42.3	1.63 (0.96–2.77)		1
23.0–27.4	44.9	40.6	2.95 (1.75–4.98)		2
≥27.5	29.9	11.9	6.19 (3.60–10.6)	<0.01	3
Parity					
≥2	43.0	38.2	1.00 (reference)		0
1	48.0	57.6	1.81 (1.45–2.27)		1
0 (nulliparous)	9.0	4.2	3.85 (2.67–5.55)	<0.01	2
Menstruation span, y					
<28.0	8.4	24.1	1.00 (reference)		0
28.0–31.9	15.7	24.5	1.55 (1.14–2.12)		1
32.0–35.1	26.1	26.2	2.23 (1.63–3.05)		2
≥35.2	49.9	24.2	4.17 (3.02–5.76)	<0.01	3
Menopausal status					
Premenopausal	43.2	53.8	1.00 (reference)		
Postmenopausal	56.8	46.2	1.50 (1.27–1.77)	<0.01	
Oral contraceptive use					
Ever	18.0	22.9	1.00 (reference)		0
Never	82.0	77.1	1.62 (1.33–1.97)	<0.01	1
Hormone replacement therapy use					
Never	95.2	96.6	1.00 (reference)		
Ever	4.8	3.4	1.18 (0.80–1.74)	0.41	
Regular physical activity					
Ever	28.2	28.8	1.00 (reference)		0
Never	71.8	71.2	1.42 (1.18–1.70)	<0.01	1
Cigarette smoking					
Ever	3.1	3.0	1.00 (reference)		
Never	96.9	97.0	1.15 (0.73–1.80)	0.56	
Alcohol use					
Ever	3.1	4.9	1.00 (reference)		0
Never	96.9	95.1	1.71 (1.13–2.59)	0.01	1
History of hypertension or diabetes					
No	56.9	81.9	1.00 (reference)		0
Yes	43.1	18.1	2.75 (2.29–3.30)	<0.01	1
Family history of endometrial or colorectal cancers					
No	94.4	97.8	1.00 (reference)		0
Yes	5.6	2.2	2.56 (1.70–3.87)	<0.01	1

* Adjusted for age (continuous) and education.

*Note*: Cut points for menstruation span are based on the quartile distribution in controls and for BMI are based on the WHO BMI cut-points for Asian populations.

In addition, we examined the effect of two independent SNPs on associations between scored established risk factors and endometrial cancer in the stratified analysis by genotype. Tests for trend were performed by entering the categorical variables as continuous parameters in the models. Multiplicative interactions between scored risk factors and genotype subgroups were assessed by comparing the difference of the log likelihoods between models with the main effects and models with both the main effects and the interaction terms. We used the Hosmer and Lemeshow goodness-of-fit test to check the logistic regression models. All statistical tests were two-tailed, and a *P* value<0.05 was considered statistically significant. *P*-values presented in this paper were not corrected for multiple tests. None of the associations for the 86 SNPs tested would reach statistical significance (5.8*10^−4^), if the Bonferroni correction were applied.

## Results

**Table 1** presents associations of the established risk factors with endometrial cancer risk in our study population. Cases were older than controls (mean age: 54.8 *vs.* 50.8 *P*<0.01). Associations for most factors presented in the Table 1 were in agreement with prior published literature. The frequency of alcohol consumption was low in our population (3.1% for cases and 4.9% for controls) and was inversely associated with endometrial cancer risk. No apparent association was identified between HRT use or cigarette smoking and cancer risk, but the rate of exposure for these factors was very low.

Associations of non-genetic risk scores with endometrial cancer risk are presented in **Table 2**. When analyzed as a continuous variable, the OR was 1.65 (95% CI: 1.56–1.75) for each increment in the risk score. When risk scores were categorized into tertiles, women who were in the highest tertile had 8.83-fold (95% CI: 6.44–12.1) higher endometrial cancer risk (*P* trend <0.01) compared with women who were in the lowest tertile. Risk estimates increased 3.76-fold among women with a risk score ≥7 (median range) compared with women whose risk score was <7.

**Table 2:** Associations of risk scores for established risk factors with endometrial cancer risk, the Shanghai Endometrial Cancer Genetics Study (SECGS).

Risk scores†	Cases *N* (%)	Controls *N* (%)	Endometrial cancer risk OR (95% CI)*
Total for all 14 scores (continuous) Categories	1,028 (100.0)	1,922 (100.0)	1.65 (1.56–1.75)
0–5	72 (7.0)	597 (31.1)	1.00 (reference)
6	108 (10.5)	342 (17.8)	2.82 (2.00–3.97)
7	143 (13.9)	332 (17.3)	3.92 (2.79–5.50)
8	205 (19.9)	319 (16.6)	5.99 (4.26–8.42)
9	224 (21.8)	214 (11.1)	9.95 (6.94–14.3)
≥10	276 (26.9)	118 (6.1)	22.5 (15.3–33.1)
			*P* trend <0.01
Tertile distribution			
<6	72 (7.0)	597 (31.1)	1.00 (reference)
6–7	251 (24.4)	674 (35.1)	3.05 (2.25–4.15)
>7	705 (68.6)	651 (33.9)	8.83 (6.44–12.1)
			*P* trend <0.01
Median range			
7	180 (17.5)	939 (48.9)	1.00 (reference)
≥7	848 (82.5)	983 (51.1)	3.76 (3.05–4.64)

* Adjusted for age (continuous) and education.

† Constructed on the basis of risk association: age (y;<45 = 0, 45–54 = 1, ≥55 = 2); BMI (<18.5 = 0, 18.5–22.9 = 1, 23.0–27.4 = 2, ≥27.5 = 3); parity (0 = 2, 1 = 1, ≥2 = 0); menstruation span (y; <28.0 = 0, 28.0–31.9 = 1, 32.0–35.1 = 2, ≥35.2 = 3); use of oral contraceptives (never = 1, ever = 0); regular physical activity (never = 1, ever = 0); alcohol consumption (yes = 1, no = 0); history of diabetes or hypertension (no = 0, yes = 1) and family history of endometrial or colorectal cancer (no = 0, yes = 1) as shown in Table 1.

*Note*: Cut offs for risk score are based on the distribution among controls.

Nine of the 72 SNPs in the *BCL2* gene (rs12961976, rs17759659, rs2170294, rs4941195, rs4987768, rs7230177, rs7231901, rs7243091 and rs9807663) had a statistically significant association with endometrial cancer risk (*P*<0.05, **Table 3**), all of which are intronic to *BCL2*. These 9 SNPs lie in 4 haplotype blocks in the *BCL2* genomic region and r^2^ values were high (>0.8) between seven of these SNPs (data not shown). SNP rs17759659 was in LD (r^2^ of 0.825) with SNPs rs10460159, rs11663788 and rs6810, which are in predicted miRNA binding sites. Overall, only 2 SNPs, rs17759659 and rs7243091, (r^2^<0.3) were found to be independent of each other and were both associated with risk of endometrial cancer, even after adjusting for each other in the model. The minor alleles (*G*) of both rs17759659 and rs7243091 were associated with increased risk of endometrial cancer (for rs17759659, OR = 1.27, 95% CI: 1.04–1.53 for women with the *GA* genotype; for rs7243091, OR = 1.82, 95% CI: 1.21–2.73 for women with the *GG* genotype). None of the SNPs in the *BAD, BAX* or *BAK1* genes were associated with risk.

**Table 3:** Association of genetic variants in BCL-2-family genes with endometrial cancer risk among 1,028 cases and 1,922 controls, the Shanghai Endometrial Cancer Genetics Study (SECGS).

Gene/chromosome				Heterozygous (Aa)	Homozygous for minor allele (aa)	Additive P trend^c^
SNP (N = 86)	Alleles^a^	Gene region	MAF^b^	OR (95% CI)	OR (95% CI)	
**BCL2/chr18**						
rs1009726	C/G	5UTR	19.1	1.08 (0.92–1.28)	1.05 (0.67–1.64)	0.41
rs10503077	C/T	5UTR	3.8	1.05 (0.78–1.40)	3.88 (0.35–43.7)	0.56
rs10503078	A/G	5UTR	37.8	0.99 (0.83–1.17)	0.96 (0.75–1.22)	0.80
rs11152370	C/G	5UTR	11.7	0.98 (0.81–1.19)	0.66 (0.32–1.34)	0.48
rs11152374	A/G	3UTR	25.7	1.09 (0.92–1.28)	1.24 (0.92–1.69)	0.12
rs11659758	A/T	5UTR	19.3	1.00 (0.84–1.18)	0.78 (0.50–1.22)	0.52
rs11872329	C/T	5UTR	3.7	1.22 (0.91–1.63)	1.96 (0.45–8.59)	0.13
rs11877911	C/G	5UTR	9.5	0.99 (0.80–1.22)	0.94 (0.42–2.08)	0.91
rs12454712	C/T	5UTR	44.6	0.95 (0.79–1.17)	0.94 (0.75–1.17)	0.74
rs12457190	A/G	5UTR	35.4	0.95 (0.80–1.12)	1.03 (0.84–1.33)	0.94
rs12457831	C/G	5UTR	35.8	0.97 (0.82–1.15)	1.03 (0.79–1.32)	0.97
rs12457893	A/C	3UTR	38.4	0.96 (0.81–1.13)	1.07 (0.84–1.34)	0.82
rs12605881	A/T	3UTR	34.4	0.93 (0.78–1.09)	1.05 (0.82–1.34)	0.92
rs12961672	C/G	5UTR	19.1	0.99 (0.84–1.17)	0.87 (0.56–1.37)	0.69
**rs12961976**	C/T	3UTR	18.2	1.16 (0.97–1.38)	1.30 (0.93–1.82)	**0.04**
rs12963776	A/G	5UTR	39.3	0.99 (0.84–1.18)	1.02 (0.80–1.30)	0.90
rs12967026	C/G	5UTR	37.9	1.16 (0.98–1.38)	1.13 (0.89–1.44)	0.16
rs1381547	C/T	3UTR	38.4	1.14 (0.96–1.35)	0.94 (0.75–1.19)	0.99
rs1381548	A/G	3UTR	26.6	0.94 (0.80–1.11)	0.75 (0.53–1.05)	0.12
rs1531697	A/T	5UTR	70.3	0.98 (0.82–1.16)	1.03 (0.81–1.30)	0.93
rs17070739	G/T	5UTR	22.7	1.03 (0.88–1.22)	1.15 (0.79–1.69)	0.46
rs17070809	A/G	5UTR	32	1.03 (0.88–1.21)	0.91 (0.69–1.20)	0.79
rs17070904	C/T	3UTR	8.8	0.96 (0.78–1.19)	0.69 (0.27–1.79)	0.54
**rs17759659**	A/G	3UTR	10.1	1.27 (1.04–1.53)	1.16 (0.53–2.53)	**0.02**
rs17841945	A/G	5UTR	12.5	0.87 (0.71–1.05)	0.77 (0.40–1.47)	0.11
rs1944419	A/T	3UTR	40.9	0.91 (0.77–1.08)	1.01 (0.80–1.26)	0.81
rs1944421	A/G	3UTR	13.1	0.96 (0.80–1.16)	1.33 (0.76–2.33)	0.88
rs1944423	A/G	Promotor	37.9	1.12 (0.95–1.33)	1.24 (0.97–1.57)	0.07
rs1982673	G/T	5UTR	43.5	0.92 (0.77–1.10)	1.03 (0.82–1.29)	0.98
rs2046135	A/T	5UTR	16.8	1.09 (0.92–1.29)	0.94 (0.55–1.60)	0.49
**rs2170294**	G/T	Intron	34.4	0.95 (0.81–1.12)	0.73 (0.56–0.95)	**0.04**
rs2199937	C/T	5UTR	48.8	1.08 (0.90–1.31)	1.13 (0.91–1.41)	0.28
rs2279115	A/C	Promotor	37.8	1.13 (0.95–1.34)	1.19 (0.94–1.52)	0.09
rs2849382	C/T	3UTR	23.2	0.93 (0.79–1.10)	1.14 (0.81–1.62)	0.92
rs2850755	C/T	3UTR	10.0	0.99 (0.81–1.22)	0.62 (0.26–1.50)	0.60
rs2850758	A/G	3UTR	10.2	1.00 (0.81–1.23)	0.63 (0.26–1.50)	0.65
rs3744933	G/T	5UTR	7.6	0.97 (0.77–1.21)	2.14 (0.91–5.05)	0.61
rs3744951	C/T	3UTR	4.5	1.13 (0.87–1.48)	2.42 (0.21–28.1)	0.29
rs4941183	A/G	5UTR	40.5	0.90 (0.76–1.07)	0.97 (0.76–1.23)	0.50
rs4941185	A/G	5UTR	48.3	1.02 (0.84–1.23)	1.02 (0.82–1.28)	0.89
rs4941188	A/G	5UTR	38.9	0.98 (0.83–1.16)	0.97 (0.77–1.24)	0.79
rs4941190	A/G	3UTR	10.1	1.08 (0.89–1.32)	1.39 (0.62–3.13)	0.30
rs4941192	A/G	3UTR	7.2	1.07 (0.86–1.34)	2.20 (0.67–7.19)	0.32
**rs4941195**	A/C	3UTR	22.6	1.28 (1.09–1.50)	1.06 (0.74–1.52)	**0.03**
rs4987716	G/T	3UTR	7.6	1.08 (0.87–1.35)	1.07 (0.43–2.64)	0.49
rs4987721	A/G	3UTR	17.7	0.88 (0.74–1.04)	1.27 (0.82–1.99)	0.54
rs4987739	A/G	3UTR	5.5	1.23 (0.97–1.57)	1.14 (0.15–8.37)	0.09
**rs4987768**	G/T	3UTR	29.5	1.16 (0.98–1.37)	1.32 (1.00–1.73)	**0.02**
rs4987808	A/C	5UTR	8.8	0.96 (0.77–1.19)	0.58 (0.21–1.64)	0.45
rs4987839	A/G	5UTR	39.7	0.93 (0.78–1.10)	0.99 (0.78–1.25)	0.70
rs6567326	G/T	5UTR	47.4	0.96 (0.80–1.15)	0.87 (0.70–1.09)	0.22
rs6567328	A/G	5UTR	35.6	0.91 (0.77–1.07)	0.93 (0.72–1.19)	0.33
rs6567334	A/C	3UTR	44.4	1.03 (0.86–1.23)	1.24 (1.00–1.54)	0.07
rs720321	A/G	5UTR	14.5	1.16 (0.98–1.37)	N/A	0.37
rs7226979	C/T	3UTR	45.6	0.94 (0.78–1.12)	1.04 (0.84–1.29)	0.83
**rs7230177**	C/T	5UTR	32.3	0.91 (0.77–1.07)	0.68 (0.51–0.90)	**0.01**
rs7230970	C/T	5UTR	25.5	1.02 (0.87–1.20)	1.18 (0.85–1.65)	0.44
**rs7231901**	A/C	3UTR	32.7	0.92 (0.78–1.08)	0.69 (0.52–0.91)	**0.01**
rs7236090	C/T	3UTR	41.5	0.92 (0.77–1.09)	1.18 (0.95–1.48)	0.28
rs7240319	A/G	5UTR	16.5	0.87 (0.73–1.04)	0.96 (0.58–1.60)	0.17
rs7240326	C/T	3UTR	24.0	0.95 (0.80–1.12)	0.87 (0.61–1.25)	0.38
**rs7243091**	A/G	5UTR	18.3	1.16 (0.98–1.37)	1.82 (1.21–2.73)	**0.003**
rs7243985	C/T	Promotor	26.5	0.96 (0.81–1.13)	1.04 (0.77–1.41)	0.90
rs8083946	A/G	3UTR	36.0	1.00 (0.85–1.18)	0.92 (0.71–1.19)	0.63
rs8084922	G/C	3UTR	33.8	0.89 (0.76–1.05)	1.07 (0.83–1.38)	0.86
rs8089331	G/C	3UTR	24.0	0.97 (0.82–1.14)	1.01 (0.72–1.40)	0.81
rs8094651	A/C	5UTR	49.3	0.95 (0.79–1.14)	0.87 (0.70–1.08)	0.20
rs8096471	A/G	3UTR	15.7	0.90 (0.75–1.08)	1.31 (0.81–2.13)	0.74
rs954954	G/T	Intron	16.1	1.16 (0.97–1.37)	1.15 (0.69–1.92)	0.10
**rs9807663**	A/T	3UTR	34.5	0.94 (0.80–1.11)	0.73 (0.56–0.95)	**0.04**
rs9955190	A/G	5UTR	23.3	0.97 (0.83–1.15)	1.11 (0.78–1.58)	0.91
rs9965844	C/G	5UTR	7.7	1.00 (0.80–1.25)	1.71 (0.71–4.11)	0.61
rs8084922	G/C	3UTR	33.8	0.89 (0.76–1.05)	1.07 (0.83–1.38)	0.86
rs8089331	G/C	3UTR	24.0	0.97 (0.82–1.14)	1.01 (0.72–1.40)	0.81
**BAD/chr11**						
rs660442	C/T	5UTR	6.6	1.12 (0.89–1.42)	0.20 (0.02–1.58)	0.69
rs1468558	C/T	5UTR	7.3	0.91 (0.72–1.15)	0.26 (0.06–1.19)	0.16
rs671976	A/G	Intron	34.7	0.85 (0.72–1.01)	1.17 (0.91–1.49)	0.84
rs477895	A/G	3UTR	23.3	0.91 (0.77–1.08)	0.85 (0.59–1.21)	0.18
**BAX/chr19**						
rs11667229	C/T	Promotor	38.5	1.14 (0.96–1.36)	0.96 (0.76–1.22)	0.79
rs11667351	G/T	Promotor	6.7	1.06 (0.84–1.33)	0.14 (0.02–1.12)	0.77
rs1805419	A/G	Intron	37.5	1.00 (0.85–1.19)	0.96 (0.74–1.23)	0.93
rs8108882	C/T	3UTR	47.0	0.98 (0.82–1.18)	1.05 (0.84–1.30)	0.73
rs905238	A/G	3UTR	35.5	0.93 (0.78–1.09)	1.04 (0.81–1.34)	0.88
**BAK1/chr6**		3UTR				
rs210132	G/T	3UTR	38.7	1.01 (0.85–1.19)	0.90 (0.71–1.15)	0.51
rs210134	A/G	3UTR	26.0	1.01 (0.86–1.19)	1.05 (0.77–1.43)	0.78
rs210139	A/C	Intron	30.8	1.13 (0.96–1.33)	1.01 (0.77–1.33)	0.46
rs563751	A/G	3UTR	47.1	1.07 (0.89–1.29)	0.92 (0.74–1.15)	0.52
rs5745577	A/G	Intron	3.9	1.04 (0.78–1.39)	1.00 (0.09–11.4)	0.79

a Major/minor alleles.

b Minor allele frequency in controls.

c Adjusted for age and education. SNPs associated with endometrial cancer risk (*P* value<0.05) are represented in bold. *P* values were not corrected for multiple tests.

*Notes*:

-*AA* indicates homozygotes for the major allele, who were used as the reference group (not shown in the table), *Aa* indicates heterozygotes and *aa* indicates homozygotes for the minor allele.

-No deviation from Hardy-Weinberg Equilibrium (HWE) was observed for any given SNP among controls.

-5UTR, 5′ untranslated region; 3′ UTR, 3′ untranslated region.

-Significant SNPs are presented in bold.

We further evaluated whether SNPs rs7243091 or rs17759659 have a modifying effect on associations between the score of established risk factors and endometrial cancer risk (**Table 4**). We found no evidence that these two SNPs modify the association of non-genetic risk factors with endometrial cancer (*P* for interaction  = 0.87 for rs7243091 and 0.62 for rs17759659).

**Table 4:** Effects of *BCL2* gene variants on associations between risk scores for established risk factors and endometrial cancer risk, the Shanghai Endometrial Cancer Genetics Study (SECGS).

Risk scores for established risk factors	Homozygous for major allele (AA)			Heterozygous or homozygous for minor allele (Aa/aa)			
	Cases	Controls	OR (95% CI)*	Cases	Controls	OR (95% CI)*	
**rs7243091**							
Continuous Tertiles	634	1,272	1.65 (1.54–1.77)	394	648	1.67 (1.52–1.84)	
<6	45	401	1.00 (reference)	27	195	1.24 (0.75–2.06)	
6–7	144	418	3.06 (2.10–4.48)	107	255	3.72 (2.50–5.45)	
>7	445	453	8.68 (5.96–12.7)	260	198	11.6 (7.79–17.3)	
*P* value for interaction = 0.87							
**rs17759659**							
Continuous Tertiles	790	1,553	1.65 (1.55–1.76)	237	369	1.64 (1.46–1.84)	
<6	58	485	1.00 (reference)	14	112	1.02 (0.55–1.90)	
6–7	188	545	2.79 (1.99–3.90)	63	129	3.93 (2.59–5.98)	
>7	544	523	8.33 (5.92–11.7)	160	128	9.90 (6.68–14.7)	
*P* value for interaction = 0.62							

* Adjusted for age (continuous) and education.

## Discussion

To the best of our knowledge, this is the first study to evaluate genetic variants in the BCL-2 family of genes together with the quantified effect of established risk factors on endometrial cancer risk. Common genetic variants in the BCL-2-family genes *BCL2*, *BAD, BAX* and *BAK1* were comprehensively evaluated for associations with endometrial cancer risk. Nine of the 72 SNPs examined in the *BCL2* gene, rs12961976, rs17759659, rs2170294, rs4941195, rs4987768, rs7230177, rs7231901, rs7243091 and rs9807663, had a statistically significantly association with endometrial cancer risk and two SNPs, rs17759659 and rs7243091, (r^2^<0.3) were found to be independent of each other and were both associated with risk of endometrial cancer. The 9 disease-associated SNPs in the intronic region of *BCL2* do not appear to alter amino acids, so their relationship to the underlying biology of endometrial cancer remains unclear. We also found that a risk score created based on established endometrial cancer-risk factors [32]–[34], including age, BMI, parity, menstrual span, physical activity, OC use, alcohol consumption, history of diabetes and hypertension and history of endometrial and colorectal cancers in first-degree relatives, was highly predictive of endometrial cancer risk in our study population. However, risk score associations with endometrial cancer were not appreciably modified by SNPs rs7243091 or rs17759659.

Despite the limited observational data on the relationship between BCL-2-gene family polymorphisms and endometrial cancer, our findings on the associations between genetic variants in the *BCL2* gene and endometrial cancer risk are consistent with findings from previous human tumor tissue studies, which have clearly demonstrated a role for members of the BCL-2 family of genes, particularly the *BCL2* and *BAX* genes, in endometrial carcinoma [11], [12], [14]–[16]. Most such studies have found low or no expression of *BCL2* in endometrial carcinoma [14], [17], [18], although the evidence was not entirely consistent [14]. Laboratory studies have also demonstrated that *BCL2* gene expression is related to the degree of aggressiveness and differentiation in endometrial carcinoma [6], [14]. For example, Vaskivuo *et al.* observed low levels of *BCL2* expression in grade I endometrial carcinomas and very low levels or no *BCL2* expression in grade II and III endometrial carcinomas, respectively [14], suggesting that the *BCL2* g ene is a valuable predictor of disease progression [15].

It has also been suggested that *BCL2* may be the most hormone-dependent member of the BCL-2 family of genes and that *BCL2* expression patterns in the normal endometrium may vary depending on the menstrual cycle phase or hormonal environment [4], [5]. This cyclical pattern of *BCL2* expression decreased or disappeared after administration of levonogestrel, a synthetic progestogen used in some hormonal contraceptives [35], and *BCL2* was over expressed in the anti-progestin-treated endometrium [36], demonstrating that exogenous steroid hormones affect *BCL2* expression in the human endometrium. However, in our study, we found no modifiable effect of two independent SNPs within the *BCL2* gene on associations between non-genetic factors, including hormonal factors, and endometrial cancer risk. These results may be due to the size of our sample not being large enough to detect such interactions or the studied SNPs may not be causally linked to BCL2 gene function. Further studies with a larger sample size, comprehensive evaluation of the genetic variants and, preferably, a direct measurement of estrogen levels are needed to understand the role of *BCL2* in endometrial carcinogenesis.

Our study has several strengths, including use of a population-based sample, a relatively large sample size, a genetically homogeneous population, high response rates at recruitment (82.8% for cases and 74.4% [the SECS] and 90.3% [the SBCS] for controls), and histopathology-confirmed case status, all of which help to limit selection and misclassification biases. Detailed information on reproductive and lifestyle factors, medical history, and measured anthropometrics were collected by trained interviewers. No epidemiologic study has yet simultaneously evaluated a large number of polymorphisms in several apoptosis-related genes in the BCL-2 family with endometrial cancer risk. In addition, we also evaluated the potential interactions between established risk factors and genetic markers. Limitations of this study should also be considered when interpreting the results. First, this study evaluated 86 SNPs, and none of the associations would reach statistical significance (5.8*10^−4^), if the Bonferroni correction were applied, probably due to the small sample size. Second, our study is the first systematic study of genetic associations between BCL-2-family genes and endometrial cancer risk, and the findings need to be replicated in an independent cohort of endometrial cancer patients. Last, the lack of a direct measurement of estrogen hormone levels prevented us from investigating the potential role of BCL-2-family genes in the context of hormone exposure.

In summary, we found that two independent SNPs in the *BCL2* gene were associated with endometrial cancer risk among Chinese women. However, we found little evidence that these polymorphisms modify the association of established risk factors with endometrial cancer. Further studies are needed to confirm our findings and to elucidate the role of gene-environment interactions in endometrial cancer development.
