# Correction: Association of Genetic Markers in the BCL-2 Family of Apoptosis-Related Genes with Endometrial Cancer Risk in a Chinese Population

**DOI:** 10.1371/journal.pone.0117632

**Published:** 2015-01-28

**Authors:** The PLOS ONE Staff

In Table 3, the minor and major alleles for several SNPs are incorrect. Please view the corrected Table 3 here.

**Table 3:** Association of genetic variants in BCL-2-family genes with endometrial cancer risk among 1,028 cases and 1,922 controls, the Shanghai Endometrial Cancer Genetics Study (SECGS).

Gene/chromosome				Heterozygous (Aa)	Homozygous for minor allele (aa)	
SNP (*N* = 86)	Alleles^a^	Gene region	MAF^b^	OR (95% CI)	OR (95% CI)	**Additive *P* trend ^c^**
**BCL2/chr18**						
rs1009726	C/G	5UTR	0.191	1.08 (0.92–1.28)	1.05 (0.67–1.64)	0.41
rs10503077	C/T	5UTR	0.038	1.05 (0.78–1.40)	3.88 (0.35–43.7)	0.56
rs10503078	G/A	5UTR	0.378	0.99 (0.83–1.17)	0.96 (0.75–1.22)	0.80
rs11152370	G/C	5UTR	0.117	0.98 (0.81–1.19)	0.66 (0.32–1.34)	0.48
rs11152374	G/A	3UTR	0.257	1.09 (0.92–1.28)	1.24 (0.92–1.69)	0.12
rs11659758	T/A	5UTR	0.193	1.00 (0.84–1.18)	0.78 (0.50–1.22)	0.52
rs11872329	C/T	5UTR	0.037	1.22 (0.91–1.63)	1.96 (0.45–8.59)	0.13
rs11877911	C/G	5UTR	0.095	0.99 (0.80–1.22)	0.94 (0.42–2.08)	0.91
rs12454712	T/C	5UTR	0.446	0.95 (0.79–1.17)	0.94 (0.75–1.17)	0.74
rs12457190	G/A	5UTR	0.354	0.95 (0.80–1.12)	1.03 (0.84–1.33)	0.94
rs12457831	G/C	5UTR	0.359	0.97 (0.82–1.15)	1.03 (0.79–1.32)	0.97
rs12457893	C/A	3UTR	0.384	0.96 (0.81–1.13)	1.07 (0.84–1.34)	0.82
rs12605881	A/T	3UTR	0.344	0.93 (0.78–1.09)	1.05 (0.82–1.34)	0.92
rs12961672	G/C	5UTR	0.191	0.99 (0.84–1.17)	0.87 (0.56–1.37)	0.69
**rs12961976**	C/T	3UTR	0.182	1.16 (0.97–1.38)	1.30 (0.93–1.82)	**0.04**
rs12963776	G/A	5UTR	0.393	0.99 (0.84–1.18)	1.02 (0.80–1.30)	0.90
rs12967026	C/G	5UTR	0.38	1.16 (0.98–1.38)	1.13 (0.89–1.44)	0.16
rs1381547	T/C	3UTR	0.384	1.14 (0.96–1.35)	0.94 (0.75–1.19)	0.99
rs1381548	G/A	3UTR	0.266	0.94 (0.80–1.11)	0.75 (0.53–1.05)	0.12
rs1531697	T/A	5UTR	0.404	0.98 (0.82–1.16)	1.03 (0.81–1.30)	0.93
rs17070739	T/G	5UTR	0.227	1.03 (0.88–1.22)	1.15 (0.79–1.69)	0.46
rs17070809	G/A	5UTR	0.32	1.03 (0.88–1.21)	0.91 (0.69–1.20)	0.79
rs17070904	C/T	3UTR	0.088	0.96 (0.78–1.19)	0.69 (0.27–1.79)	0.54
**rs17759659**	A/G	3UTR	0.101	1.27 (1.04–1.53)	1.16 (0.53–2.53)	**0.02**
rs17841945	G/A	5UTR	0.125	0.87 (0.71–1.05)	0.77 (0.40–1.47)	0.11
rs1944419	T/A	3UTR	0.409	0.91 (0.77–1.08)	1.01 (0.80–1.26)	0.81
rs1944421	T/C	3UTR	0.131	0.96 (0.80–1.16)	1.33 (0.76–2.33)	0.88
rs1944423	A/G	Promotor	0.379	1.12 (0.95–1.33)	1.24 (0.97–1.57)	0.07
rs1982673	A/C	5UTR	0.435	0.92 (0.77–1.10)	1.03 (0.82–1.29)	0.98
rs2046135	T/A	5UTR	0.168	1.09 (0.92–1.29)	0.94 (0.55–1.60)	0.49
**rs2170294**	C/A	Intron	0.344	0.95 (0.81–1.12)	0.73 (0.56–0.95)	**0.04**
rs2199937	T/C	5UTR	0.489	1.08 (0.90–1.31)	1.13 (0.91–1.41)	0.28
rs2279115	G/T	Promotor	0.378	1.13 (0.95–1.34)	1.19 (0.94–1.52)	0.09
rs2849382	A/G	3UTR	0.233	0.93 (0.79–1.10)	1.14 (0.81–1.62)	0.92
rs2850755	A/G	3UTR	0.101	0.99 (0.81–1.22)	0.62 (0.26–1.50)	0.60
rs2850758	T/C	3UTR	0.1	1.00 (0.81–1.23)	0.63 (0.26–1.50)	0.65
rs3744933	G/T	5UTR	0.076	0.97 (0.77–1.21)	2.14 (0.91–5.05)	0.61
rs3744951	T/C	3UTR	0.045	1.13 (0.87–1.48)	2.42 (0.21–28.1)	0.29
rs4941183	A/G	5UTR	0.405	0.90 (0.76–1.07)	0.97 (0.76–1.23)	0.50
rs4941185	G/A	5UTR	0.483	1.02 (0.84–1.23)	1.02 (0.82–1.28)	0.89
rs4941188	A/G	5UTR	0.39	0.98 (0.83–1.16)	0.97 (0.77–1.24)	0.79
rs4941190	A/G	3UTR	0.103	1.08 (0.89–1.32)	1.39 (0.62–3.13)	0.30
rs4941192	A/G	3UTR	0.072	1.07 (0.86–1.34)	2.20 (0.67–7.19)	0.32
**rs4941195**	A/C	3UTR	0.226	1.28 (1.09–1.50)	1.06 (0.74–1.52)	**0.03**
rs4987716	C/A	3UTR	0.076	1.08 (0.87–1.35)	1.07 (0.43–2.64)	0.49
rs4987721	T/C	3UTR	0.177	0.88 (0.74–1.04)	1.27 (0.82–1.99)	0.54
rs4987739	C/T	3UTR	0.055	1.23 (0.97–1.57)	1.14 (0.15–8.37)	0.09
**rs4987768**	C/A	3UTR	0.295	1.16 (0.98–1.37)	1.32 (1.00–1.73)	**0.02**
rs4987808	G/T	5UTR	0.088	0.96 (0.77–1.19)	0.58 (0.21–1.64)	0.45
rs4987839	T/C	5UTR	0.397	0.93 (0.78–1.10)	0.99 (0.78–1.25)	0.70
rs6567326	G/T	5UTR	0.474	0.96 (0.80–1.15)	0.87 (0.70–1.09)	0.22
rs6567328	A/G	5UTR	0.356	0.91 (0.77–1.07)	0.93 (0.72–1.19)	0.33
rs6567334	A/C	3UTR	0.444	1.03 (0.86–1.23)	1.24 (1.00–1.54)	0.07
rs720321	G/A	5UTR	0.145	1.16 (0.98–1.37)	N/A	0.37
rs7226979	T/C	3UTR	0.456	0.94 (0.78–1.12)	1.04 (0.84–1.29)	0.83
**rs7230177**	C/T	5UTR	0.323	0.91 (0.77–1.07)	0.68 (0.51–0.90)	**0.01**
rs7230970	T/C	5UTR	0.255	1.02 (0.87–1.20)	1.18 (0.85–1.65)	0.44
**rs7231901**	A/C	3UTR	0.327	0.92 (0.78–1.08)	0.69 (0.52–0.91)	**0.01**
rs7236090	T/C	3UTR	0.415	0.92 (0.77–1.09)	1.18 (0.95–1.48)	0.28
rs7240319	A/G	5UTR	0.165	0.87 (0.73–1.04)	0.96 (0.58–1.60)	0.17
rs7240326	T/C	3UTR	0.24	0.95 (0.80–1.12)	0.87 (0.61–1.25)	0.38
**rs7243091**	G/A	5UTR	0.183	1.16 (0.98–1.37)	1.82 (1.21–2.73)	**0.003**
rs7243985	C/T	Promotor	0.265	0.96 (0.81–1.13)	1.04 (0.77–1.41)	0.90
rs8083946	A/G	3UTR	0.36	1.00 (0.85–1.18)	0.92 (0.71–1.19)	0.63
rs8084922	C/G	3UTR	0.338	0.89 (0.76–1.05)	1.07 (0.83–1.38)	0.86
rs8089331	C/G	3UTR	0.24	0.97 (0.82–1.14)	1.01 (0.72–1.40)	0.81
rs8094651	C/A	5UTR	0.493	0.95 (0.79–1.14)	0.87 (0.70–1.08)	0.20
rs8096471	G/A	3UTR	0.157	0.90 (0.75–1.08)	1.31 (0.81–2.13)	0.74
rs954954	A/C	Intron	0.161	1.16 (0.97–1.37)	1.15 (0.69–1.92)	0.10
**rs9807663**	T/A	3UTR	0.345	0.94 (0.80–1.11)	0.73 (0.56–0.95)	**0.04**
rs9955190	A/G	5UTR	0.233	0.97 (0.83–1.15)	1.11 (0.78–1.58)	0.91
rs9965844	G/C	5UTR	0.078	1.00 (0.80–1.25)	1.71 (0.71–4.11)	0.61
**BAD/chr11**						
rs660442	G/A	5UTR	0.066	1.13 (0.89–1.42)	0.20 (0.02–1.59)	0.69
rs1468558	G/A	5UTR	0.073	0.91 (0.72–1.15)	0.26 (0.06–1.19)	0.16
rs671976	A/G	Intron	0.347	0.86 (0.73–1.01)	1.17 (0.91–1.49)	0.84
rs477895	T/C	3UTR	0.233	0.91 (0.77–1.08)	0.85 (0.59–1.21)	0.18
**BAX/chr19**						
rs11667229	T/C	Promotor	0.385	1.15 (0.97–1.36)	0.97 (0.76–1.23)	0.79
rs11667351	T/G	Promotor	0.067	1.06 (0.84–1.33)	0.14 (0.02–1.12)	0.77
rs1805419	G/A	Intron	0.375	1.00 (0.85–1.19)	0.96 (0.74–1.23)	0.93
rs8108882	T/C	3UTR	0.47	0.98 (0.82–1.18)	1.04 (0.84–1.30)	0.73
rs905238	A/G	3UTR	0.355	0.93 (0.78–1.09)	1.05 (0.81–1.34)	0.88
**BAK1/chr6**						
rs210132	G/T	3UTR	0.387	1.01 (0.85–1.19)	0.90 (0.71–1.15)	0.51
rs210134	G/A	3UTR	0.26	1.01 (0.86–1.19)	1.05 (0.77–1.43)	0.78
rs210139	C/A	Intron	0.308	1.13 (0.96–1.33)	1.01 (0.77–1.33)	0.46
rs563751	A/G	3UTR	0.471	1.07 (0.89–1.29)	0.93 (0.74–1.15)	0.52
rs5745577	C/T	Intron	0.039	1.04 (0.78–1.39)	1.00 (0.09–11.4)	0.80
